# Memory clinics in context

**Published:** 2009-01

**Authors:** David Jolley, Esme Moniz-Cook

**Affiliations:** Honorary Reader Manchester University, PSSRU Dover Building, Dover Street, M13 9PL, Hull HU3 2 SG; 1 Professor of Clinical Psychology and Ageing, University of Hull, Institute of Rehabilitation c/o Coltman Street Day Hospital, Coltman Street, Hull HU3 2 SG

**Keywords:** Memory Clinics, dementia, memory services, psycho-geriatric, services, psycho-social interventions

## Abstract

The growing number of older people in all parts of the world raises the question of how best to respond to their health needs, including those associated with memory impairment. Specialist Memory Clinics have a role to play, complementing community services which reach out to older people with mental health problems and encompassing younger people who become forgetful. Dementia is the most common syndrome seen, but there are other important treatable conditions which present with subjective or objective dysmnesia. Memory Clinics provide a high quality, devoted focus for early intervention, treatment, support and research.

## INTRODUCTION

Memory clinics as a means of providing help to people with dementia and other memory-related disorders reflect a North American and European tradition of centralizing expertise at an out-patient hospital-based service. In some ways they ran counter to the philosophy and practice of the psychogeriatric movement which transformed mental health services for older people in the UK from the late 1960s[1–3] when, two decades since its conception, the UK National Health Service had begun to struggle with its mission to provide good quality care to all, free at the point of entry and regardless of age, gender, class or economic standing. The discipline of psychogeriatric medicine emerged from a combination of the attributes of geriatric medicine and social psychiatry, which were adapted to the special characteristics and needs of older people with mental health problems.[4,5] What was new at the time was the shift towards taking specialist expertise out of the hospital to the most severely distressed group of older people at their familiar, home-based settings. The aim was to provide care and alleviate suffering in both patients and carers with minimal recourse to mental hospital care, which had little to commend it.

Memory clinics in the UK developed some 20 years later, first appearing in the mid 1980’s[6,7] to attract people with memory problems as early as possible to the best available expertise, thus increasing the numbers of people who were offered specialized help outside mental institutions. Their cost was seen as equivalent to just four long term ‘continuing care’ mental hospital beds.[7] Their aims were to: ‘forestall deterioration by early diagnosis and treatment; identify and treat disorders other than dementia; evaluate new therapeutic agents and; reassure people who in the absence of morbid deficits are worried’.[6–8] They have done much to develop medical and neuropsychological approaches to individuals and families, contributed extensively to biological, pharmacological, and neuropsychological research and helped to take forward understanding of the characteristics, aetiology, and natural history of the dementias. However, apart from a few exceptions[9–11] this has to date been at the expense of developing population based services with a public health ethic where active actions to promote health and well-being of the population can be seen.

## CURRENT STATUS AND DISTRIBUTION

Memory clinics are now found worldwide[12–41] and as the movement has matured, clinical and research interest groups or ‘networks’ have developed, to disseminate best practice or argue the case for sustained growth, even where finance is limited.[30,42] In the Netherlands, such groups are beginning to generate schedules to facilitate description and comparison of activities and quality standards.[43] Clinics in the UK are mostly, but not always[7,44] established within the orbit of mental health services, but this is not so elsewhere in the world, where general medicine, neurology, geriatric medicine, and other disciplines can take the lead.[7,13,34,44–49] Some patient groups such as those with learning disabilities,[50] early onset dementia[51] and those with particular language or cultural needs[52] require special consideration, whilst urban and rural communities may benefit from differentiated, tailored approaches.[11,38,53] The memory clinic movement is therefore not one of the uniformity but can vary in terms of setting, patient characteristics and the types or balance of activities undertaken. These in turn have an impact on relationships with other agencies and on outcomes, including their role in long-term support and follow through.

## WHAT IS DONE IN MEMORY CLINICS?

Texts outlining the structure, function and procedures that are used in memory clinics are growing.[54,28,35,48,55–66] Despite their lack of homogeneity, most clinics have a similar structure of a core multi-professional team who work from a clinic base where out-patient sessions are arranged at specified times. Their function is to provide assessment and early interventions. Many also act as vehicles of best practice innovations with wider functions such as providing information, resources, co-ordination, liaison, signposting, teaching, and research.

## STRUCTURE

**The team:** This is always led by a clinician or clinicians, often organized by a senior nurse, supported by secretarial time. There is need to organize paper work, transport, investigations and to gather supplementary information and answer questions which may come from many sources and at any time. Multi-disciplinary assessment work may be focused on particular days, leaving time between for providing intervention, educational activity, personal study and to make contact with other experts or support agencies, whose help may be useful in individual situations.

Specialist input comes from:

- Medical staff: Physician, neurologist or psychiatrist, trainees, non-consultant career grade doctors or physician assistants may be involved. It is a good training venue for students.
- Clinical psychologists: The lead psychologist may be the clinic director and may be supported by colleagues, assistants, trainees, and students.
- Nursing staff: Often provide the main source of continuity for patients and families and forge and maintain links with other agencies.
- Occupational therapists: have particular skills in supporting rehabilitation by translating the understanding of individual difficulties into a program of therapy or provision of prostheses.
- Alzheimer’s society: Representation of the local branch of the society can be available on a regular basis within clinics. This has multiple advantages in easing families toward sources of information and education provided by the society and also encouraging them to join local supportive group meetings.
- Other staff, less often seen as core team members, include social work, speech therapy, dieticians, and clinical pharmacists.
- Referral arrangements for particular needs such as chiropody, dentistry, hearing and eyesight clinics, translation, benefits, and legal matters, are often in place with some of these available as ‘in house’ service specifications.
- Some clinics promote links with faith communities and alternative therapists for aroma therapy, art therapy, or acupuncture.

**A place and time:** The clinic’s base should have at least two rooms to interview both patient and carer separately if needed. They should also be accessible to the population served and well equipped: rooms require furniture, heating, lighting, and communications and some staff need special equipment. Ideally they should be quiet with minimal distraction. Larger rooms can be used for staff to discuss their findings, agree plans, and share these with patients and carers. Communal areas can be used to receive and welcome patients and families. Refreshments should be available. Toilets are essential and facilities need to take account of physical disability of patients or carers. Getting to and from the clinic may require special transport arrangements.

Suites of rooms such as this may be available in health centers, general hospitals, community hospitals, day hospitals or other premises.[7,9,41,67] It may be necessary to make for the less than perfect accommodation to enhance accessibility of location. There is much to be said for taking the skills of the memory clinic to primary care or other community settings, simply to be effectively available and to minimize stigma and inconvenience to patients and carers.[7,9,10,67] A specialist tier of hospital based clinics can support such ‘travelling/community memory clinics’[11] and there is a place for super-specialist third tier clinics as centers of research and the investigation and treatment of very complex cases.

In practice most memory clinics provide their full multi-professional interactive clinical presence on one or two days each week. Key members of clinic staff remain involved with memory clinic activity throughout the working week, but others are busy elsewhere. An answer-phone, with response from key staff at the beginning of each working day is helpful in maintaining communication over the out-of-hours and weekend periods and is reassuring to all parties.

## FUNCTION: ASSESSMENT

**Medical:** Everyone developing a memory problem requires a full review of their general health, including history, physical examination, consideration of medication, and investigations to establish a diagnosis and clarify all contributing factors.[68,69] This may be undertaken by the referring doctor in primary care though it may be necessary to add to this within the clinic. All patients require a full review of their mental health: history and mental state examination. Dementia is often complicated by ‘non-cognitive’ symptoms of mood, perception, belief or behavior,[69] but depression and other disorders may in themselves be the basis of subjective and objective problems of cognition.

**Psychological and functional:** Psychological assessment is undertaken by a number of disciplines with the support and supervision of a clinical psychologist.[68] Clinical assessments are usually complemented by validated instruments which measure aspects of cognition and non-cognitive function.[47,54,58–60,64,70] Dementia, particularly in older people, is not a difficult condition to diagnose in most instances and the simplest, shortest schedules are as effective as longer, more complex ones.[68] However, more detailed serial use of neuropsychological schedules may be necessary to achieve a sound differential diagnosis with people very early in the course of a dementia or for complex presentations. In addition, these structured tests reflect more sensitively particular strengths and weaknesses exhibited by an individual[64,69,70,71–73] which can be helpful in rehabilitation.[61] Loss of function may be subtle and relatively minor, as in failure to remember details of a shared conversation or television programme viewed together. However, even this can be irritating and undermining. At the extreme, dementia may make it impossible for the individual to get in or out of clothing, find their way in a familiar home or make a simple meal or drink. Between these extremes, there is a massive range of ‘need – does’ for the maintenance of an autonomous dignified adult life. A number of valid and reliable scales have been used although all have their limitations.[74] Codifying, measuring, and communicating changes in function is arguably the most important activity of a memory clinic.[61,75,76]

**Social:** Basic information about each patient’s social circumstances will be collected as a matter of routine within clinics.[68] People with memory impairment are compromised and become dependent on others. Families and other informal carers keep them well and safe.[77] It is important to be aware who is doing what and at what cost: there may be gaps in the supportive network, or clues that failures could occur.[68] Carers sometimes have health problems of their own or responsibilities to others.[78–80]

**Other considerations:** Spiritual aspects of healthcare are now receiving the attention they deserve.[81] Dementia influences and is influenced by the spiritual and faith lives of patients and carers.[82] This is an important consideration for faith communities, where older people are respected and often very active. Clinics are beginning to appreciate these matters and some are making appropriate responses.

Preserved cognition is necessary to manage monies, prepare a will, consent to medical treatment, or enter into contracts relating to accommodation or marriage. It is necessary if the individual is to be deemed fit to drive a motor vehicle.[83] Laws relating to such matters are being upgraded around the world and clinics are in a strong position to provide advice and in such matters.

## FUNCTION: TREATMENT AND REHABILITATION

**Medical interventions:** Assessment may reveal treatable physical or psychiatric pathology.[69] Appropriate therapy may be offered from the clinic or result in referral to other experts. When using psychotropic agents with tenuous cognition in this age group and with tenuous cognition, it is always important to beware of the possibility of adverse side-effects.[84]

The licensing of the cholinesterase inhibitors and memantine as moderately useful and safe treatments for Alzheimer’s disease encouraged optimism and brought more people to ask for help. In the UK, the prescription of these compounds is restricted by guidance from the National Institute of Clinical Excellence (NICE). This has had the benefit of requiring that patients are competently assessed before treatment by a memory clinic or its equivalent and that they are followed up.[45,79,85] Lewy body dementia also responds fairly well to cholinesterase inhibitors and so is managed in a similar way. Vascular dementia requires thorough investigation, treatment, and follow-up of vascular pathology, often in association with other experts, but mixed vascular/Alzheimer’s presentations are common and may benefit from a cholinesterase inhibitor.

**Psychological interventions and other therapies:** Diagnostic disclosure programmes are a key intervention in memory clinic settings.[61,68,86] Additionally clinical psychologists work with individuals, families and groups to minimize potential problems associated with failing cognition and to encourage activities which will maintain cognition, autonomy, pleasure, and quality of life. Approaches include: cognitive stimulation, cognitive rehabilitation, reminiscence therapy, emotion-based care and adaptations from standard psychological therapies such as cognitive behavior therapy (CBT) which can be used with the person, carer or with both.[68,87] Other approaches include pet, music, exercise and art therapies[69,87] and the alternative therapies. These include herbal remedies and aromatic oils,[88] massage, multi-sensory stimulation, and aromatherapy.[69]

**Social interventions and support for carers:** People with dementia are hugely supported by their families. This is confirmed in analyses from the UK and the rest of Europe, as well as North America. It is even more evident in less affluent countries.[89,90] The work of the family can be supplemented by professional agencies: Social services, the voluntary or independent sector.[91–93] This may involve shared care at home, day care or respite admissions and may give way eventually to admission to a care home. For people still at home there is growing interest in the use of new technology to complement traditional personal care systems.[94] Clinics have a role in identifying appropriate local facilities which may be suitable for particular family situations. In addition, memory clinics should organize regular activities for carers and patients to satisfy their needs for up to date information and education and to share in a supportive network with others experiencing similar difficulties.[95]

**Liaison with other agencies:** Memory clinics are a component of a wider system of multi-agency care. Inevitably, there is overlap in what units do. In sharing information about individuals a balance is needed which respects privacy but champions openness, minimizing duplication but avoiding omissions by assuming ‘someone else will do it’. The primary care team is likely to know the patient, family, and other local resources better than other agencies and may be in the best position to coordinate services.[67,96,97] Patient-held records and practice-based memory clinics are helpful in this and in sustaining a vision of always seeking to maintain or recover best function when ‘cure’ is not achievable.[67,68,87,97,98]

**Education and training:** Clinics are often involved in the training of undergraduate and postgraduate students of any or all of the professions which contribute to their work. Key to their strength in this is the contribution which comes from patients and families. There remains troubling evidence of the need to improve knowledge amongst people delivering services in the community, in residential and nursing homes and in hospitals. Clinics can and should aim to allocate time to offering outreach education to improve the care and experiences of patients and families: their funding and commissioning should reflect this.[91,99]

**Health promotion:** Much is now understood about the factors associated with the development and progress of dementia. Many risk factors for dementia are shared with risks for vascular disease. This means that programmes of Health Education designed to help individuals and communities to improve their general health will reduce the risk of developing dementia.[97,100–102] Clinics attract a depth and breadth of knowledge which equips staff to contribute usefully to such programs.[11,68,97]

**Research and audit:** Memory clinics have encouraged and practiced research and audit from their beginnings and in all situations.[6,7,9,15,45,59,62,65,103–108] Their disciplined collection of data and maintenance of good records at referral and in follow up, not only in focused, funded projects, but as a feature of routine practice, is a major strength and resource. Much has been made of this, though there is more which could be achieved.

**Follow up/follow through to death and beyond:** Though the original remit of clinics was to reach out to people with early evidence or fears of failing memory, they have become so much part of the infra structure of services and respect and trust in the quality of their knowledge so great, that many are now contributing to the full spectrum of care and aftercare of people with dementia. This may be a direct involvement with individuals and families, or be indirect through the local community service. Some clinics relate to an admiral nurse[108] thus contributing their expertise in support of better long-term care, including care in dying, and the support of families in the life which follows.[109]

## WHAT IS ACHIEVED BY MEMORY CLINICS?

There is no doubt that clinics encourage earlier detection of dementia and related conditions.[16,24,26,29,63,74,19,108,110] They is one important factor in breaking down barriers to care.[15,68,111] General practitioners praise their assessments, investigation and diagnosis and communications about these and patients and carers are also complimentary.[16,112–116] However, ongoing support to manage developing difficulties and advice about future care options is not so secure.[16–19,113,116–118] Many people come to clinics with very limited knowledge of dementia and what they might expect from their assessment and most fear prognosis.[118,119] Thus, time and a preparedness to go over matters again and again are essential.[68,86,87,97] One simple and inexpensive way to improve communication is to copy clinical letters to patients and families.[120]

## MEMORY CLINICS IN CONTEXT – A REFLECTION

At present roughly six percent of the world’s population is aged 65 years or older: three percent in Africa, five percent in South East Asia and Latin America, 16 percent plus in parts of Europe. Within countries there are concentrations of older people produced when survival patterns are complicated by differential migration of young and old. Until recently, most interest has been taken in old age in the developed world of Europe, North America, Japan, and Australasia. Yet two-thirds of the world’s elderly live in the third world and roughly six percent of the world’s current population is aged 65 years or older. This average has extremes of low numbers (three percent in Africa, five percent in South East Asia and Latin America) to higher numbers (16 percent and more) in parts of Europe. Within countries there are communities with concentrations of older people, products of survival compounded by migration of old people into the community or migration of younger people away from it.[93,121,122] The proportion of the third world’s elderly population is set to increase rapidly with improvements in life expectation.[123] The shift toward more very old people (where dementia incidence and prevalence are highest) will mean the number of people with dementia will rise impressively: Prince and his colleagues estimated there to be 24.3 million people with dementia 2005 a new case coming into being every seven seconds.[93,123,124]

The main response to the growing needs of the dementia population will be, as now, within families and local (primary) health care.[96] The 10/66 group began an ambitious programme to bolster local care with improved knowledge and instruction in effective care techniques.[92,125–127] Non-medical primary health care dementia workers will strengthen primary health care input[41,96,97,128,129] and organize training in effective, evidence-based psycho-social interventions.[87] This is a logical and economic way to deal with the situation, and has similarities to the early years of psychogeriatrics in the UK. Indeed, it has much to commend still as an approach which might be taken worldwide. Within this framework one can foresee primary care (first tier) memory clinics being established as travelling/community outreach ventures from hospital-based clinics in centers of excellence.[11,15,68,97] This will ensure as wide a population coverage as possible, ensure that all advantage is taken of local strengths in support and care systems and yet provide access to expert investigation and advice for the most difficult and complex cases when necessary. Quality of service and the opportunity to be involved in audit, research, and research networks will also be encouraged by this system.[15,130]
